# Efficacy of single-dose cholecalciferol in the blood pressure of patients with type 2 diabetes, hypertension and hypovitaminoses D

**DOI:** 10.1038/s41598-020-76646-6

**Published:** 2020-11-12

**Authors:** Tatiana P. de Paula, Juliano S. R. Moreira, Luiza F. Sperb, Maria Elisa P. Muller, Thais Steemburgo, Luciana V. Viana

**Affiliations:** 1 Endocrine Division, Hospital de Clínicas de Porto Alegre, Universidade Federal do Rio Grande do Sul, Rua Ramiro Barcelos, 2350, Prédio 12, 4º andar, Porto Alegre, RS 90035-003 Brazil; 2 Postgraduate Program in Food, Nutrition, and Health, Universidade Federal do Rio Grande do Sul, Porto Alegre, Brazil

**Keywords:** Endocrinology, Health care

## Abstract

Observational and experimental data reinforce the concept that vitamin D is associated with the pathogenesis of arterial hypertension. We investigated the effect of a single dose of 100,000 IU of cholecalciferol, in office blood pressure (BP), and 24-h ambulatory blood pressure monitoring (ABPM) in patients with type 2 diabetes mellitus (DM), hypertension, and hypovitaminosis D. Forty-three patients were randomized to a placebo or cholecalciferol group. BP was assessed by office measurements and 24-h ABPM, before and after intervention. At week 8, a greater decrease in median ABPM values was observed in cholecalciferol supplementation than in the placebo group for systolic 24-h (− 7.5 vs. − 1; *P* = 0.02), systolic daytime (− 7 vs. − 1; *P* = 0.007), systolic nighttime (− 7.0 vs. 3; *P* = 0.009), diastolic 24-h (− 3.5 vs. − 1; *P* = 0.037), and daytime DBP (− 5 vs. 0; *P* = 0.01). Office DBP was also reduced after vitamin D supplementation. A single dose of vitamin D_3_ improves BP in patients with type 2 diabetes, hypertension, and vitamin D insufficiency, regardless of vitamin D normalization. Vitamin D supplementation could be a valuable tool to treat patients with type 2 DM, hypertension, and hypovitaminosis D.

**Trial registration**: Clinicaltrials.gov NCT 02204527.

## Introduction

Observational and experimental data reinforce the concept that vitamin D is associated with the pathogenesis of arterial hypertension^1–5^. The precise mechanism by which vitamin D lowers blood pressure (BP) is still unknown. However, effects on endothelial function, inflammation and oxidative stress as well as reduced activity of the renin–angiotensin–aldosterone system (RAS)^5,6^ and reduced levels of parathyroid hormone have been proposed with mechanism of action of vitamin D.

In hypertensive patients with type 2 diabetes mellitus (DM) and hypovitaminosis D the potential beneficial effects of vitamin D supplementation are still scarce^7–9^. Some of the more recent Mendelian randomization studies suggest the influence of low vitamin D concentration on blood pressure^10^. However, previous meta-analyses showed mixed effects on BP with supplementation of vitamin D in adults^7–9^. Nevertheless, some of these meta-analyses included studies that examined the effects of different types of vitamin D (1-α-hydroxylated vitamin D derivatives or calcitriol, paricalcitol; and ergocalciferol or cholecalciferol), as well as a different access (oral, intramuscular, and parenteral vitamin D)^8^.

We hypothesized that the anti-hypertensive effect of vitamin D_3_ could be stronger in patients with vitamin D insufficiency. In fact, improved serum 25(OH) D concentrations in hypertensive individuals who had insufficient vitamin D were associated with improved control of systolic and diastolic BP and conferred a significant risk reduction for hypertension^4,5^. Indeed a meta-analysis that evaluates only vitamin D supplementation in vitamin D deficient subjects showed a small but significant reduction of diastolic BP^11^.

This study was performed in a population where effects are supposed to be maximal, namely in patients with hypertension and vitamin D insufficiency. The present trial was designed to determine the effect of a single dose of vitamin D_3_ compared to a placebo on BP values, independent of vitamin D_3_ normalization, evaluated by office and ambulatory blood pressure monitoring (ABPM), in patients with type 2 DM, hypertension, and hypovitaminosis D after 8 weeks of supplementation.

## Methods

This article was designed and reported according to the Consort 2010 Statement^12^ providing all sections suggested to parallel-group randomized trials.

### Trial design

In an 8-week, randomized double-blind, parallel, placebo-controlled clinical trial, patients were randomly assigned to a single dose of 100,000 IU of vitamin D_3_ or a matching placebo by an online computer-generated randomly permutated codes (www.randomization.com).

The outcome of the study was changes in office and ABPM measurements. The study was carried out between October 2015 and December 2016 and was conducted in accordance with the guidelines established in the Declaration of Helsinki^13^. The Hospital Ethics Committee of the Hospital de Clinicas de Porto Alegre (Porto Alegre, Brazil) approved the protocol, and all patients gave their written informed consent. This clinical trial was registered at clinicaltrials.gov as NCT02204527 in 30/07/2014.

### Participants

Outpatients with type 2 DM (HbA1c 6.5–10%), hypertension (office systolic BP ≥ 140 mm Hg or diastolic ≥ 90 mm Hg or ongoing antihypertensive treatment)^14,15^ and hypovitaminosis D (25(OH) D serum concentration below 20 ng/mL or 50 nmol/L) were recruited to participate in the study at the Endocrine Division of Hospital de Clinicas in Porto Alegre, Brazil. Exclusion criteria were the following: pregnant or lactating women, oral calcium or vitamin D supplementation or any medications affecting calcium or vitamin D metabolism (estrogens and calcitonin), change of antihypertensive treatment (drugs or lifestyle modifications) in the previous 4 weeks or planned changes of antihypertensive treatment during the study, any other concomitant clinical disease that could influence vitamin D metabolism (e.g. renal, hepatic, other endocrinologic disorders, and malignancies), body mass index (BMI) > 40 kg/m^2^, creatinine > 2 mg/dL (or > 176 mmol/L), and an inability or unwillingness to participate.

### Study protocol

**Figure 1:**
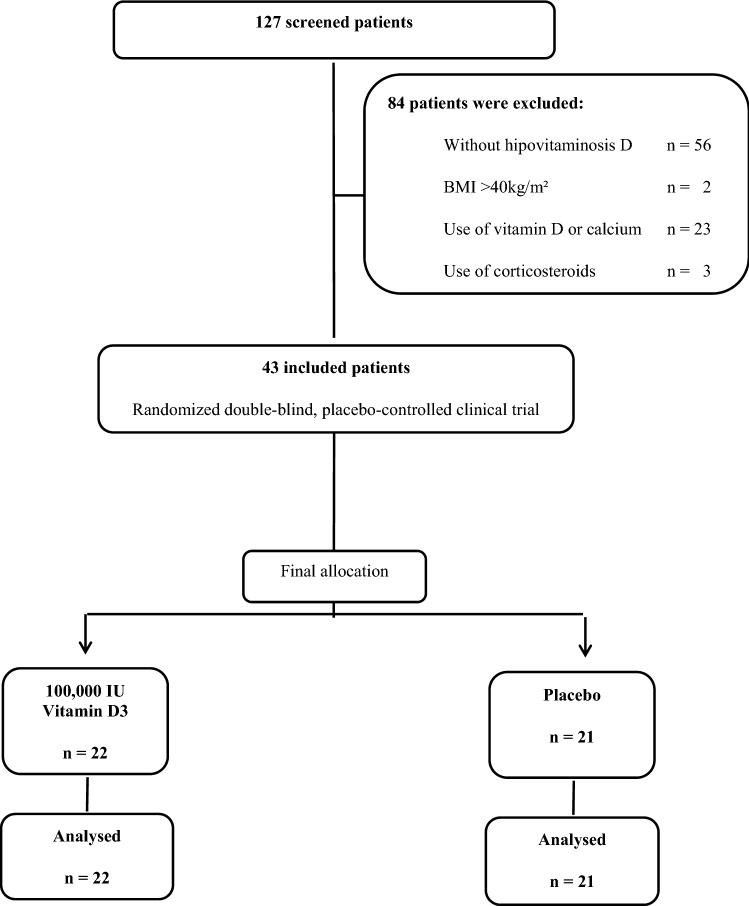
Flow diagram showing the included patients.

Figure 1 shows the flowchart of the study protocol. Of 127 screened patients, 84 were excluded: 56 without hypovitaminosis D, 23 in use of vitamin D, calcium or corticosteroids and 2 with BMI > 40 kg/m^2^. A total of 43 participants were randomized, of whom 100% attended the 8 weeks follow-up visits.

Selected patients were fully informed about the study, signed the consent form, and underwent a 2-week run-in period that involved two office visits. Office BP was measured at each visit. Subjects were advised not to change their lifestyle during the study. Antihypertensive drugs were also not modified during the study. Baseline laboratory, clinical, physical activity, and nutrition evaluations were performed, and all patients underwent 24-h ABPM. After the run-in period, participants were randomly assigned to one of two parallel groups: (1) a placebo group with capsules containing microcrystalline cellulose and (2) an intervention group with capsules with 100,000 IU of cholecalciferol (25(OH)D). Participants ingested the medication in the presence of the researchers to ensure 100% compliance. Office BP, ABPM measures and clinical, physical activity, and nutrition evaluations were performed at the end of the study. The intervention period was 8 weeks.

Medication (50,000 IU Cholecalciferol; Vitamin D_3_; Addera™, Anapólis, Goias, Brazil) and placebo were bottled and labeled by Quinta Essência pharmaceuticals, and participants and researchers were blinded until the end of the study including the statistical analyses. All concomitant medications were kept unchanged to prevent possible effects on the study parameters.

### Outcomes

#### Blood Pressure

The changes in ABPM measurements were the primary outcome, and changes in office systolic and diastolic BP were secondary outcomes. A change in BP was the difference between BP at the baseline and at the end of the study (8 weeks).

Blood pressure was measured at the office and by 24-h ABPM. Office BP was measured twice at each visit in at least 2-min intervals, in the sitting position after 5 min of resting (on a chair with feet on the floor and the arm supported at the heart level)^15^, and the mean value was used for analysis (Omron Automatic BP Monitor HEM-705CP, Omron Healthcare, Inc, Lake Forest, IL). Patients were advised to avoid caffeine, exercise, and smoking 30 min before the measurements. ABPM was measured at baseline and at the end the study (week 8). ABPM was performed by an oscillometry method using a Spacelabs device (Spacelabs Healthcare, Snoqualmie, WA 90,207, serial numbers 207-054280, 207-024751, 207-054290, 207-056568, and 207038016, with calibration certification), with a 15-min interval during the day and a 20-min interval during the night^14–16^. All ABPM measurements were obtained on a normal workday. Sleep time was recorded as the period between the time when the patient went to bed and the time when the patient woke up the next morning. The means of 24-h, daytime, and night-time systolic and diastolic BP, BP loads (percentage of 24-h and daytime BP measurements ≥ 140/90 mm Hg and night-time BP measurements ≥ 120/80 mm Hg) were recorded, as well as pulse pressure (PP) (systolic BP – diastolic BP). Nondipping was defined as the failure of the BP to fall by at least 10% during sleep^14,16^. The night-time/daytime BP ratios for systolic and diastolic BP were calculated by dividing the night-time by the daytime BP values. The BP status of the patients was classified according to ABPM measurements as dippers: N/D BP ratio ≤ 0.90; and non-dippers: N/D BP ratio > 0.90^17^.

### Clinical evaluation, nutritional and physical activity assessment

Clinical and demographic data were collected based on standard protocol, and medical examination procedures were performed. Briefly, we collected data about type 2 DM and hypertension duration, smoking, alcohol intake, ethnic self-classification, current medications, and usual sunlight exposure on routine days. A detailed description of the nutritional and physical activity assessment of this trial has been published elsewhere^18^.

### Laboratory measurements

Blood samples were collected after at least an 8-h fast, and the season of the year was recorded. A detailed description of plasma glucose, sodium, HbA1c, total cholesterol, high-density lipoprotein cholesterol (HDL-cholesterol), low-density lipoprotein cholesterol (LDL-cholesterol), triglycerides, and urinary 24-h albumin, calcium, and sodium was determined and has been published elsewhere^18^. Vitamin D (25(OH)D) and the parathyroid hormone (PTH) were determined by a chemiluminescence technique.

### Statistical analysis

The estimated number of included patients (N = 43) was based on a 7.3 mm Hg reduction in office systolic BP with *2 SD, *a power of 80% and an α of 0.05, after a single dose of 100,000 IU of cholecalciferol in patients with type 2 DM^19^. Twenty-one participants would be required in each group to achieve a power of 80% and an α of 0.05.

Results were expressed as mean (SD), median (P25–P75), or number of patients with the characteristic (percentage). Student t-tests, Mann–Whitney U tests, and Pearson chi-square tests were used as appropriate. All data analyses were performed using the statistical software package IBM SPSS version 20.0 (Chicago, IL), and the type I error rate was fixed at *P* < 0.05 (two-tailed). The *P* values of less than 0.05 were assumed to be significant.

### Randomization

The researchers prepared a computer-generated randomization code (1:1) stored in sealed dark envelopes until the end of the study. Each participant was given two pills of medication or placebo in sequence to preserve allocation concealment. Pills were identical in presentation and participants and researchers were blinded until the end of the study including the statistical analyses for each group to which they were allocated.

## Results

Figure 1 shows the flow diagram of the study protocol. Out of 127 screened patients (between October 2015 and December 2016), 84 were excluded, mainly due to normal vitamin D (n = 56), vitamin D or calcium supplementation (n = 23), and other (n = 4). Detailed characteristics of these patients are published elsewhere^18^.

All patients who received the intervention completed the protocol and were included in the final analyses. Baseline demographic, clinical, anthropometric, and laboratory characteristics of the participants according to the intervention and placebo groups are shown in Table 1. Mean age was 65 ± 9 years old, diabetes duration was 12 ± 8 years, BMI was 31 ± 4 kg/m2, 25(OH)D 14 ± 5 ng/ml, and HbA1c was 7.6 ± 1.0% (60 mmol/mol). Eighteen patients (42%) were classified as having resistant hypertension.

**Table 1:** Baseline characteristics of studied patients.

Baseline characteristics	Vitamin D Group	Placebo Group	*P*
N	22	21	–
Male/female, No	6/16	9/12	–
Age, y	66 ± 8	65 ± 11	0.8*
White ethnicity, No. (%)	21 (96)	19 (91)	0.5^†^
Current smoking, No. (%)	2 (9)	0 (0)	0.3^c^
Diabetes duration, y	13 (6–15)	12 (5–20)	0.7*
Hypertension duration, y	17 (10–26)	20 (9–29)	0.5*
Level of Education, y	10 (8–12)	9 (8–11)	0.6*
Current alcohol intake, No. (%)	7 (32)	8 (38)	0.8^†^
Previous Cardiovascular Event No. (%)	1 (5)	2 (10)	0.5^†^
Diabetic retinopathy, No. (%)*	6 (29)	4 (18)	0.3^†^
Sunscreen use No. (%)	7 (32)	4 (19)	0.3^†^
Winter/summer season No. (%)	19(85)/ 3(14)	18(86) / 3 (14)	0.7^†^
**Blood pressure parameters**			
Office systolic BP, mm Hg	149 ± 18	147 ± 17	0.7*
Office diastolic BP, mm Hg	83 ± 7	84 ± 14	0.7*
24-h systolic ABPM, mm Hg	135 ± 12	131 ± 12	0.3*
24-h diastolic ABPM, mm Hg	75 ± 11	74 ± 7	0.6*
Daytime systolic ABPM, mm Hg	136 ± 13	135 ± 12	0.8*
Daytime diastolic ABPM, mm Hg	79 ± 12	76 ± 7	0.5*
Nighttime systolic ABPM, mm Hg	128 ± 11	124 ± 12	0.3*
Nighttime diastolic ABPM, mm Hg	69 ± 9	68 ± 8	0.8*
**Hypertension medication**			
ACE inhibitors n (%)	11 (52)	10 (48)	0.6^†^
Beta blockers n (%)	6 (27)	9 (43)	0.2^†^
ARBs, n (%)	9 (41)	8 (38)	0.6^†^
Diuretics, n (%)	16 (73)	17 (81)	0.4^†^
Calcium Channel Blockers, n (%)	4 (18)	8 (38)	0.1^†^
**Diabetes medication**			
Sulfonylureas, n (%)	22 (100)	21 (100)	1.00^†^
Biguanides, n (%)	10 (46)	8 (38)	0.4^†^
Insulin, n (%)	4 (18)	5 (24)	0.5^†^
Other, n (%)	1 (5)	0 (0)	0.5^†^
**Laboratory parameters**			
25(OH)D, ng/ml	14 ± 5	14.5 ± 4.3	0.6*
UAE 24-h No. (%)	13 (4–37)	15 (4–35)	0.7^‡^
Urinary sodium, mEq/24 h	175 ± 71	201 ± 42	0.2*
Urinary calcium, mEq/24 h	78 (44–118)	70 (42–122)	0. 4^‡^
Glycated hemoglobin, %	8 ± 1	8 ± 1	0.5*
Fasting glucose, mg/dL	150 ± 51	151 ± 57	1.0*
Total cholesterol, mg/dL	179 ± 40	183 ± 43	0.8*
HDL cholesterol, mg/dL	48 ± 9	54 ± 16	0.1*
Non-HDL Cholesterol mg/dL	131 ± 42	129 ± 42	0.9*
Triglycerides, mg/dL	147 (49–313)	109 (68–296)	0.4^‡^
LDL cholesterol, mg/dL	99 ± 39	100 ± 40	0.9*
Calcium mg/dL	9 (9–10)	10 (9–10)	0.5^‡^
Creatinine mg/dL	0.9 ± 0.2	0.8 ± 0.3	0.9*
Parathyroid hormone pg/mL	53 (44–77)	60 (47–78)	0.5^‡^
**Body parameters**			
Body mass index, kg/m^2^	31 ± 5	30 ± 4	0.8*
Waist circumference, male, cm	105 ± 10	107 ± 6	0.5*
Waist circumference, female, cm	107 ± 16	100 ± 5	0.4*
Fat mass, % (bioimpedance)	38 ± 9	39 ± 7	0.7*

y, years; ABPM, ambulatory blood pressure monitoring; ACE, angiotensinconverting enzyme; ARB, angiotensin receptor blocker; BP, blood pressure; UAE, urinary albumin excretion. Previous cardiovascular event = myocardial infarction and stroke. Sunscreen use = daily use of sunscreen on face and arms.

Data are expressed as number (%); mean (SD) and median (P25–P75).

*t test; ^†^Pearson’s chi-square; ^‡^Mann–Whitney U.

### Vitamin D and BP measurements during the study

Both treatment and placebo groups had improvements in 25(OH)D after 2 months of protocol, but the placebo group remained with hypovitaminosis at the end of the study (25(OH)D 14 ± 5 to 23 ± 7, *P* = 0.02 and control group 15 ± 5 to 19 ± 5, *P* = 0.6). At the end of the study, 11 (52.4%) subjects continued to have hypovitaminosis in the placebo group in contrast with only 4 (18%) subjects in the intervention group (*P* = 0.02). Fasting glucose and glycated hemoglobin did not change throughout the study (*P* > 0.05).

**Figure 2:**
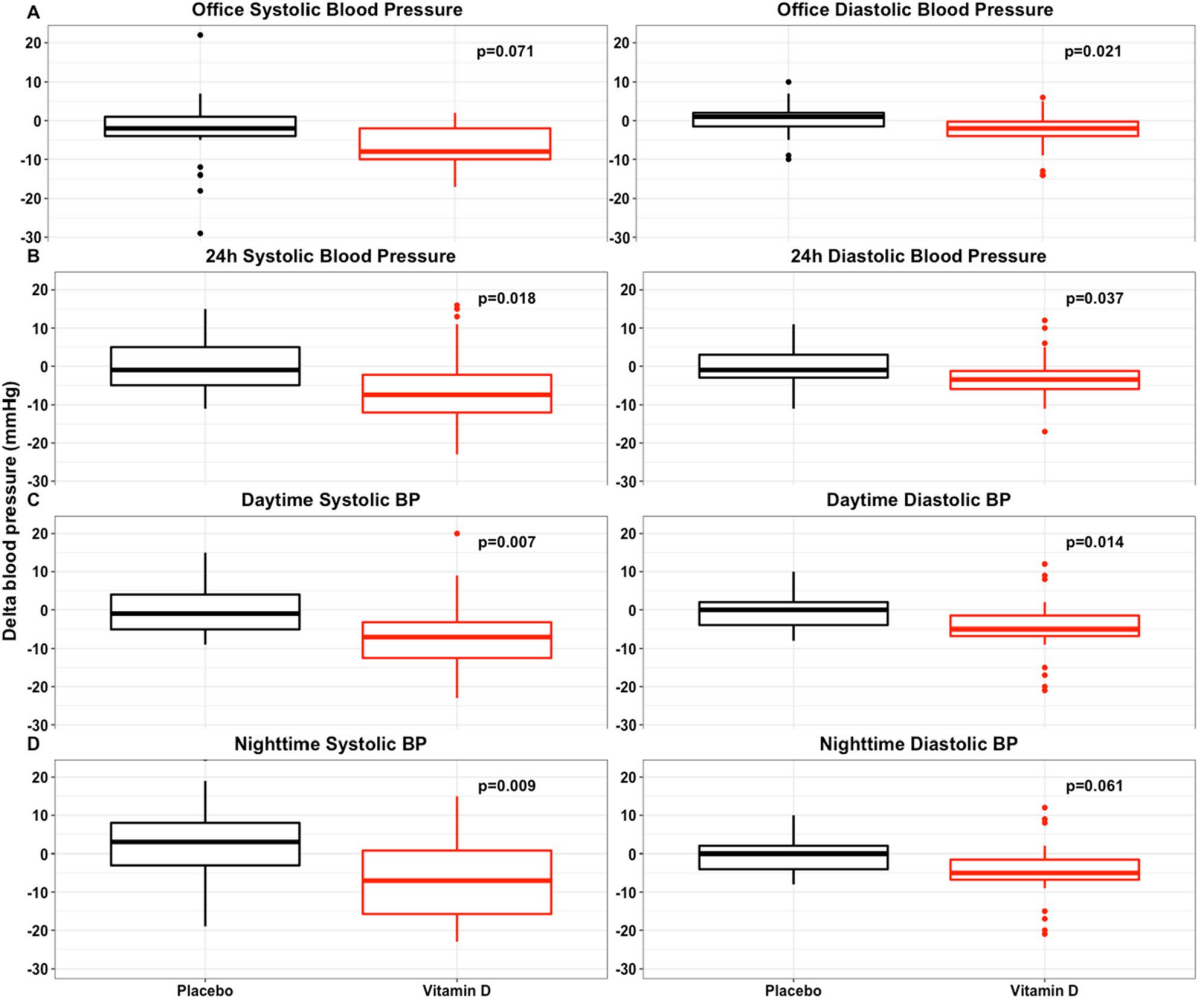
Systolic and diastolic blood pressure (BP) differences for (**A**) office BP, (**B**) ABPM 24 h BP (**C**) ABPM daytime BP and (**D**) ABPM nighttime BP. The BP delta (end-of-study minus baseline) is shown as box plots (median is the line within the box, whiskers are 10th and 90th percentiles, the points above and below indicate outliers). Black identifies placebo and red identifies vitamin D group.

Figure 2 shows the changes in BP parameters during the study. Office diastolic BP [− 2.0 (− 4.0; 0.1) vs. 1 (− 2; 2) mm Hg; *P* = 0.02] decreased in the treatment group, and there was a similar trend in systolic BP [− 8 (− 10; − 2) vs. − 2 (− 5; 2) mm Hg; *P* = 0.07] in comparison with the placebo group. Regarding ABPM changes, there was a significant reduction in systolic 24-h [− 7.5 (− 12; − 0.5) vs. − 1 (− 6; 5) mm Hg, *P* = 0.02], daytime [− 7 (− 13; − 2) vs. − 1(− 5; 6) mm Hg; *P* = 0.007], and nighttime [− 7.0 (− 17; 1) vs. 3 (− 3; 10) mm Hg; *P* = 0.009] BP. Diastolic BP at 24 h [− 3.5 (− 6; − 0.8) vs. − 1 (− 3; 3.5) mm Hg; *P* = 0.04] and in daytime [− 5.0 (− 7.5; − 0.8) vs. 0.0 (− 4; 2) mm Hg; *P* = 0.01)] was also reduced in the intervention group.

At the end of study, 65% of patients in the intervention group reached values of 24-h BP < 130/80 mm Hg as compared with 35% in the control group (*P* = 0.047). At the beginning of the study, only 26% of patients were dipping; however, at the end of the study, the intervention group dipped more [10 (46%) vs. 3 (14%); *P* = 0.03] than the control group. There was a significant reduction in 24 h pulse pressure in the vitamin D group (60 ± 9 to 57 ± 9 mmHg; P = 0.046). No serious adverse events were reported during the trial. Twenty-one percent reported a muscle and joint improvement in the intervention group as compared with 4.7% in the control group (*P* = 0.04).

## Discussion

We demonstrate that a single dose of cholecalciferol improves BP in a short period of supplementation in patients with type 2 DM with hypertension and 25(OH)D < 20 ng/ml, regardless of vitamin D_3_ normalization.

In this sample of patients with type 2 DM, hypertension, and 25(OH)D < 20 ng/ml, the administration of a single dose of cholecalciferol resulted in clinically significant decreases in BP. The most relevant effects were observed in ABPM measurements, and decreases were observed in 24-h systolic (− 7.5 mm Hg), daytime systolic (7 mm Hg), and nighttime systolic (− 7 mm Hg) BP. The magnitude of observed BP reduction seems to be clinically relevant, and this reduction is similar to that observed with antihypertensive medications^20^. Furthermore, at the end of study, 65% of the patients in the intervention group reached values lower than 130/80 mm Hg in daytime ABPM, and nocturnal dipping was also more frequent in the intervention group.

As far as we know, this is the first study to evaluate ABPM readings after vitamin D supplementation in patients with type 2 DM, hypertension, and hypovitaminosis D. ABPM has been considered to be the reference standard for the diagnosis of hypertension, allowing a complete assessment of BP parameters: 24hour, daytime, and nighttime BP means and loads; nocturnal dipping patterns; and presence of masked and white-coat hypertension. ABPM is a better predictor of future cardiovascular events as compared with conventional office-based BP measurements^21–23^. A recent study confirmed that 24-h systolic ABPM was more strongly associated with all-cause and cardiovascular mortality than the office systolic BP^24^.

Nondipping is associated with microvascular complications in type 2 DM patients, like an increase in albuminuria and more rapid progression of diabetic kidney disease^25,26^. After vitamin D supplementation, more patients had dipping patterns than in the control group. Nondipping of BP is common in patients with DM^26^, and improvement in the dipping pattern with vitamin D supplementation seems to be a promising intervention in this population, in the same way as other strategies have already proven to be effective, such as diuretics and reduction in salt intake^27^.

The exact mechanism by which vitamin D lowers BP is still unknown. Vitamin D may be an inverse endocrine regulator of the renin-angiotensin system (RAS). Experimental studies suggest that cholecalciferol suppresses the renin system^5,6^; consequently, the inappropriate activation of the RAS could increase BP and the risk of cardiovascular diseases^28^. Based on that, some authors attribute the failure of previous trials to show antihypertensive effects of vitamin D to the concomitant use of renin-angiotensin inhibitors^19,29^. However, in our trial, 48.8% of the patients were using this class of medication, and despite that, 65% of the patients in the intervention group reached the recommended goals for daytime BP^30^.

It is possible that different populations have diverse responses to the anti-hypertensive effect of vitamin D. Most studies were performed in selected white populations^19,29^ or cultures that are not regularly exposed to sunlight^31,32^. Phenotypic analyses of a Mendelian randomization study demonstrated that people with genetic variants associated with low endogenous production of 25(OH)D have an increased risk of hypertension^10^. Brazilians have a mixed ethnic background that may be favorable to the antihypertensive response of vitamin D, but genetic tests were not performed in our sample.

Vitamin D directly regulates PTH hormone secretion, since this vitamin controls dietary calcium absorption. Even a slight vitamin D insufficiency is compensated by an increase in serum PTH. A meta-analysis that evaluated vitamin D_3_ supplementation according to subgroups of remeasured serum 25(OH)D on cardiovascular and glucometabolic surrogate markers suggested that vitamin D supplementation has a beneficial effect on PTH. On the other hand, a recent systematic review that evaluated patients with hyperparathyroidism and hypovitaminosis D, observed that vitamin D replacement did not modify PTH levels while serum 25(OH)D levels were improved^33^. In our study, baseline PTH was similar in both groups, and we believe that eight weeks is a short period of time to change this hormone. In fact, in patients with secondary hyperparathyroidism from severe vitamin D deficiency, parathyroid hyperplasia may take months to over a year to reverse to normal^34^. Our study was not designed to evaluate the independent effects of PTH on BP or any mechanism of action of vitamin D_3_. We believe this a pioneering study that evaluated the effect of vitamin D_3_ only in insufficient patients, thus it is a proof of concept study of the action of this vitamin in this population.

There are important strengths in our study. The first one is the design that guarantees 100% compliance, as medication was taken in the presence of the investigator, and the second is the full scope of BP evaluation. Furthermore, there was no change in the office BP during the run-in period, excluding a possible Hawthorne effect in our sample, and no antihypertensive medication was changed during the study, avoiding other medication bias.

A possible limitation of this study is the short follow-up period: although we demonstrated an important improvement in BP after vitamin D supplementation, the beneficial effects of this intervention were only evaluated in an 8-week period. It is unknown if this effect would endure for longer periods and if it will be sustained after reaching vitamin D sufficiency for longer periods. Longer and larger clinical trials properly addressing this question in patients with type 2 DM and hypertension, as well as the capability of vitamin D to reduce hard cardiovascular outcomes, are needed.

In conclusion, a single dose of vitamin D_3_ improves BP in patients with type 2 DM, hypertension, and vitamin D insufficiency, regardless of vitamin D normalization. Since the values of vitamin D are usually low in such patients^18,19^, the supplementation of vitamin D may be part of the therapeutic arsenal in this scenario.

## Supplementary information

Supplementary Information 1.
