# Supplementary material for: Efficacy of single-dose cholecalciferol in the blood pressure of patients with type 2 diabetes, hypertension and hypovitaminoses D
Source: Sci Rep. 2020 Nov 12;10:19611. doi: 10.1038/s41598-020-76646-6 (PMC7665034; doi:10.1038/s41598-020-76646-6)
Supplement: Supplementary file 1 — Supplementary Information 1. [file 41598_2020_76646_MOESM1_ESM.pdf]

Efficacy of single-dose cholecalciferol in the blood pressure of patients with type 2 diabetes, hypertension and hypovitaminoses D

Authors:

Tatiana P de Paula, RD PhD[1], Juliano Moreira MD[1], Luiza F Sperb, MD[1] Maria Elisa P Muller, MD[1], Thais Steemburgo, RD PhD[2], Luciana V Viana, MD PhD[1]

Supplementary Table 1. Change in Office BP, ABPM, and Metabolic Parameters in Vitamin D or Placebo Groups.

Parameter	Change with Vitamin D	Change with Placebo	*P*
**Office Blood Pressure (mm Hg)**			
Office systolic BP	-8.0 (-10; -1.8)	-2.0 (-4.5; 1.5)	0.07*
Office diastolic BP	-2.0 (-4.3; .1)	1.0 (-1.8; 2)	0.02*
**Ambulatory Blood Pressure Measurements – ABPM (mm Hg)**			
24-h systolic ABPM	-7.5 (-12.0; -.5)	-1.0 (-5.5; 5.0)	0.02*
24-h diastolic ABPM	-3.5 (-6.3; -.8)	-1.0 (-3.0; 3.5)	0.04*
Daytime systolic ABPM	-7.0 (-13.0; -2.3)	-1.0 (-5.0; 5.5)	0.007*
Daytime diastolic ABPM	-5.0 (-7.5; -.8)	.0 (-4.0; 2.0)	0.01*
Nighttime systolic ABPM	-7.0 (-17.0; 1.3)	3.0 (-3; 9.5)	0.009*
Nighttime diastolic ABPM	-4.0 (-9.0; 1.3)	2.0 (-3.5; 4.5)	0.06*
**Clinical and Laboratory Parameters**			
25(OH)D	8.0 (5.9 ; 13.4)	5.0 (.8; 7.0)	0.005*
Glycated hemoglobin, %	-.1 (-.4 ;.4)	-.2 (-.3; .4)	0.7*

Abbreviations: BP, blood pressure; ABPM, ambulatory blood pressure measurements.
Data are expressed as median (P25-P75). * = *U test*
